# Organization and Evolution of *Drosophila* Terminin: Similarities and Differences between *Drosophila* and Human Telomeres

**DOI:** 10.3389/fonc.2013.00112

**Published:** 2013-05-10

**Authors:** Grazia D. Raffa, Giovanni Cenci, Laura Ciapponi, Maurizio Gatti

**Affiliations:** ^1^Istituto Pasteur-Fondazione Cenci Bolognetti, Sapienza Università di RomaRoma, Italy; ^2^Dipartimento di Biologia e Biotecnologie “C. Darwin,” Sapienza Università di RomaRoma, Italy; ^3^Istituto di Biologia e Patologia Molecolari del CNR, Sapienza Università di RomaRoma, Italy

**Keywords:** telomere protection, terminin, non-terminin proteins, telomere fusion, *Drosophila*

## Abstract

*Drosophila* lacks telomerase and fly telomeres are elongated by occasional transposition of three specialized retroelements. *Drosophila* telomeres do not terminate with GC-rich repeats and are assembled independently of the sequence of chromosome ends. Recent work has shown that *Drosophila* telomeres are capped by the terminin complex, which includes the fast-evolving proteins HOAP, HipHop, Moi, and Ver. These proteins, which are not conserved outside *Drosophilidae* and closely related Diptera, localize and function exclusively at telomeres, protecting them from fusion events. Other proteins required to prevent end-to-end fusion in flies include HP1, Eff/UbcD1, ATM, the components of the Mre11-Rad50-Nbs (MRN) complex, and the Woc transcription factor. These proteins do not share the terminin properties; they are evolutionarily conserved non-fast-evolving proteins that do not accumulate only at telomeres and do not serve telomere-specific functions. We propose that following telomerase loss, *Drosophila* rapidly evolved terminin to bind chromosome ends in a sequence-independent manner. This hypothesis suggests that terminin is the functional analog of the shelterin complex that protects human telomeres. The non-terminin proteins are instead likely to correspond to ancestral telomere-associated proteins that did not evolve as rapidly as terminin because of the functional constraints imposed by their involvement in diverse cellular processes. Thus, it appears that the main difference between *Drosophila* and human telomeres is in the protective complexes that specifically associate with the DNA termini. We believe that *Drosophila* telomeres offer excellent opportunities for investigations on human telomere biology. The identification of additional *Drosophila* genes encoding non-terminin proteins involved in telomere protection might lead to the discovery of novel components of human telomeres.

Telomeres are nucleoprotein complexes at the ends of eukaryotic chromosomes that serve at least two essential functions. They allow the cell to distinguish natural chromosome termini from broken DNA ends preventing checkpoint activation and end-to-end fusion; they cope with the inability of DNA polymerase to replicate the DNA of chromosome ends (reviewed in Jain and Cooper, 2010; O’Sullivan and Karlseder, 2010). In most organisms, the end replication problem is solved by telomerase that mediates the addition of short GC-rich repeats to chromosome ends. In *Drosophila*, telomerase is absent and telomeres are elongated by targeted transposition of specialized retroelements to chromosome ends. As a consequence, *Drosophila* telomeres do not terminate with GC-rich repeats and are assembled in a sequence-independent fashion (reviewed in Mason et al., 2008; Pardue and Debaryshe, 2011; Raffa et al., 2011; Zhang and Rong, 2012). Due to the peculiar features of its telomeres *Drosophila* has been perceived for many years as an unsuitable model system to understand human telomere biology. As a consequence, the study of fly telomeres is restricted to only a few research groups. However, recent work indicates that *Drosophila* and human telomeres are not as different as generally thought. Here we review the current achievements on *Drosophila* telomere organization and function, highlighting similarities and differences with human telomeres.

## Regulation of *Drosophila* Telomere Length

*Drosophila* telomeres are elongated by targeted transposition of three specialized non-long terminal repeat (LTR) retrotransposons called *HeT-A, TART*, and *TAHRE* (collectively abbreviated as HTT). Retrotransposition occurs through an RNA intermediate and each transposition event leads to an increase in the copy number of the element. The HTT elements transpose independently of each other only to chromosome ends with no sequence specificity for the attachment site, and target individual telomeres at rates ranging from 10^−2^ to 10^−4^ per fly generation. However the terminal elements loose approximately 70 bp at each fly generation due to replication-dependent loss of telomeric DNA. Thus, *Drosophila* telomere length homeostasis depends on the balance between the frequency of transposition events and replication-dependent terminal DNA shortening. As a result, *Drosophila* chromosomes terminate with HTT arrays of variable length, in which transposable elements are always arranged head-to-tail with the 3′ end of the most proximal element attached to the end of the chromosome (reviewed in Mason et al., 2008; Pardue and Debaryshe, 2011; Zhang and Rong, 2012).

*TART* and *TAHRE* encode both a GAG protein and a Pol protein with reverse transcriptase (RT) activity; *HeT-A* harbors a *gag* gene but does not contain an RT-coding gene, and must therefore rely on an RT encoded by another element. Telomere targeting of the HTT elements depends at least in part on the GAG proteins they encode. The transcripts of the HTT elements associate with these GAGs, which in turn associate with interphase telomeres facilitating targeted transposition. Although it lacks an RT-coding sequence, *HeT-A* is the most abundant element at *Drosophila* telomeres. Consistent with this finding, the *HeT-A* GAG specifically associates with telomeres and mediates telomeric localization of *TART* and *TAHRE* GAGs, which do not have intrinsic abilities to target chromosome ends (Pardue and Debaryshe, 2011).

Transposition of HTT elements is regulated in several ways. Mutations in *Su(var)205* and *Z4* (or *pzg*) increase the rate of *HeT-A* transcription leading to telomere elongation; *Su(var)205* and Z4 encode HP1 and a zinc finger protein involved in chromatin remodeling, respectively (Savitsky et al., 2002; Perrini et al., 2004; Silva-Sousa et al., 2012). *HeT-A* transcription is also increased by mutations in *prod*, which specifies a protein enriched in heterochromatin and at the HTT array. However, mutations in *prod* do not increase the length of the HTT arrays, suggesting that increased *HeT-A* transcription does not necessarily result in increased transposition (Török et al., 2007). In contrast, *HeT-A* transcription is negatively regulated by mutations in the JIL-1 kinase-coding gene (Silva-Sousa et al., 2012).

Drosophila telomere length is also regulated by two genes, *E(tc)* and *Tel*, that are not yet characterized at the molecular level and by the Ku70/Ku80 complex. Dominant mutations in the *E(tc)* and *Tel* cause dramatic elongations of the HTT arrays of all telomeres. *E(tc)* enhances terminal gene conversion with no effect on HTT transposition; the mechanism of *Tel* action has not been determined (Melnikova and Georgiev, 2002; Siriaco et al., 2002; Capkova Frydrychova et al., 2008). The Ku70/Ku80 complex appears to modulate the accessibility of transposition intermediates to chromosome ends, as reduction of Ku70/Ku80 activity increases telomere length without affecting HTT transcription (Melnikova et al., 2005). In the female germline, the HTT expression is negatively regulated by Piwi-interacting RNAs (piRNAs). Mutations in genes that disrupt the piRNAs pathway such as *spn-E* and *aub* result in a dramatic increase in the levels of *HeT-A* and TART transcripts (Savitsky et al., 2006; Shpiz et al., 2011).

## *Drosophila* Telomeres are Epigenetically Determined Structures

The concept of telomere was first conceived by Muller (1938). He observed that rearranged chromosomes lacking the terminal regions could not be recovered from irradiated *Drosophila* males. He thus postulated the existence of a special structure at chromosome ends, the telomere, which is essential for chromosome transmission (Muller, 1938). Subsequent studies showed that terminally deleted chromosomes (TDCs) could be recovered from irradiated females homozygous for mutations in the *mu2* gene. These TDCs were ending with different DNA sequences and were transmitted for many generations without reacquiring HTT elements. In addition, they were subject to replication-dependent erosion of terminal DNA. Thus, it is clear that TDCs terminate with highly variable DNA sequences and that they are capped by a functional neotelomere formed independently of the presence of HTT elements. The neotelomeres of TDCs appear to have the same properties and contain the same proteins as normal telomeres formed at ends of the HTT arrays (reviewed in Mason et al., 2008; Rong, 2008; Raffa et al., 2011). However, although TDCs are regularly capped, they can eventually reacquire HTT elements at their ends (Biessmann and Mason, 1988; Biessmann et al., 1990).

Terminally deleted chromosomes with neotelomeres have also been recovered from mutational events occurred in the male germline. These events include mobilization of P elements inserted near the telomere (Tower et al., 1993), breakage of dicentric chromosomes during anaphase (Ahmad and Golic, 1998; Titen and Golic, 2010), and induction of an enzymatic cut in a P element construct inserted in the telomere region (Gao et al., 2010; Beaucher et al., 2012). A recent analysis of *de novo* telomere formation at double strand breaks (DSBs) generated by the enzymatic cut method showed that neotelomere formation occurs rather frequently in wild type males and is facilitated by partial disruption of DNA repair functions such as those of Mu2/MDC1, Rad51, ATRIP, Nbs, or ATM (Beaucher et al., 2012). These results, in agreement with those obtained with *mu2* females, suggest that defects in the DNA damage response (DDR) machinery lead to persistence of DSBs allowing more time for *de novo* telomere assembly at the broken chromosome ends (Dronamraju and Mason, 2009; Beaucher et al., 2012). To reconcile these findings with Muller’s results, it has been proposed that the different experimental outcomes were due to differences in the mode of DSBs induction. In Muller’s experiments, DSBs were induced in sperm and transmitted to the embryo where *de novo* telomere formation is inefficient. In contrast, endonuclease cuts were induced in mitotic compartments of spermatogenesis where neotelomere assembly seems to be rather efficient (Ahmad and Golic, 1998; Titen and Golic, 2010; Beaucher et al., 2012).

In summary, abundant evidence demonstrates that the HTT elements are not required for the assembly and maintenance of a functional telomere. In addition, the fact that the receding ends of different TDCs have the ability to form a telomere indicates that *Drosophila* telomeres assemble in a sequence-independent fashion (reviewed in Mason et al., 2008; Rong, 2008; Raffa et al., 2011).

## Human Telomere Capping

In organisms with telomerase, telomeres are capped by protein complexes that specifically interact with the DNA repeats generated by telomerase. Human telomeres are protected by a six-protein complex (TRF1, TRF2, POT1 TPP1, TIN2, and hRap1) termed shelterin. TRF1 and TRF2 specifically bind the TTAGGG duplex, and POT1 binds the G-overhang. TIN2 and TPP1 do not directly bind DNA but interconnect TRF1/TRF2 with POT1, linking the telomere duplex with the single stranded G-overhang; TRF2 also binds hRap1, a distant homolog of *S. cerevisiae* Rap1. The shelterin subunits share three properties that distinguish them from the non-shelterin telomere-associated proteins. They are specifically enriched at telomeres; they are present at telomeres throughout the cell cycle; and their functions are limited to telomere maintenance (reviewed in Palm and de Lange, 2008). Shelterin-like elements are found in *S. pombe* and plants but not in *S. cerevisiae* or *Drosophila* (reviewed in Jain and Cooper, 2010; Raffa et al., 2011).

Although the shelterin subunits form a single six-subunit complex, deletions of individual shelterin components result in different phenotypes. Loss of TRF2 activates ATM signaling and the NHEJ DNA repair pathway leading to telomeric fusions (TFs). Depletion of either POT1 or TPP1 activates the ATR kinase and causes NHEJ-mediated TF formation (reviewed in Palm and de Lange, 2008). In contrast, loss of TRF1 activates ATR/ATM signaling and disrupts telomere replication (Martinez et al., 2009; Sfeir et al., 2009). Telomeres lacking the entire shelterin complex can be processed by six different DNA repair pathways, leading to a telomere phenotype that recapitulates the phenotypes observed after loss of the individual shelterin components (Sfeir and de Lange, 2012).

Human telomeres are also associated with the RPA-like CST complex. CST (Cdc13-Stn1-Ten1) is the major capping complex in *S. cerevisiae*. The Stn1 and Ten1 subunits of the CST complex are conserved in *S. pombe*, plants, and humans. However, the human CST complex does not share the shelterin properties and appears to have a relatively minor role in telomere capping (reviewed in Jain and Cooper, 2010). Recent work has shown that CST is required for telomere replication and G-overhang maturation (Gu et al., 2012; Stewart et al., 2012; Wang et al., 2012).

## *Drosophila* Telomere Capping

**Figure 1:**
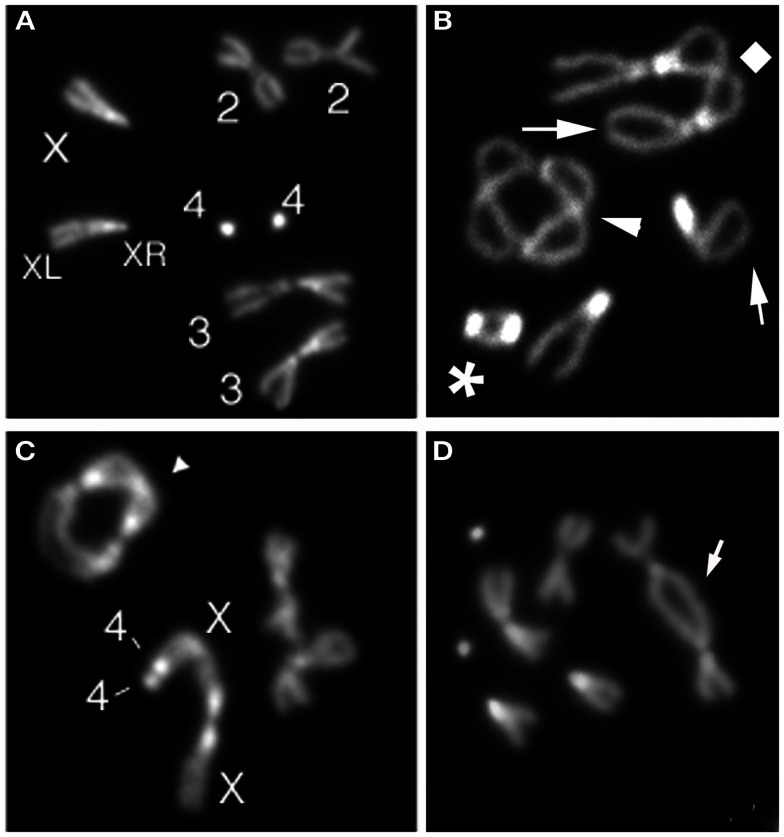
**Examples of telomeric fusions observed in *Drosophila* larval brains**. **(A)** Control (Oregon R) female metaphase with a pair of chromosomes 3, 2, and 4 and two X chromosomes (XR and XL indicate short and long X chromosome arm, respectively). **(B)** Metaphase showing a 4–4 double telomere attachment (DTA; asterisk), a 2–2 dicentric ring chromosome (arrowhead), and a 3–3 DTA (diamond). **(C)** Metaphase with a multicentric chromosome generated by a 4–4-XLXR-XR DTA and a 3–3 dicentric ring (arrowhead). **(D)** Metaphase with a dicentric chromosome (arrow) containing a 2–2 DTA.

Most of the proteins required for *Drosophila* telomere protection were identified by molecular cloning of genes specified by mutations causing TFs in larval brain cells (Figure 1). Ten genes that prevent telomere fusions (TF genes) have been so far identified by this approach (Table 1). Mutations in *caravaggio* (*cav*)*, modigliani* (*moi*), and *verrocchio* (*ver*) cause very high frequencies of TFs (∼5 per cell) and produce multicentric chromosomes that resemble little “trains” of chromosomes. The names of these genes reflect this phenotype, as three Italian trains are dubbed with the names of these famous artists. *cav* encodes HP1/ORC-associated protein (HOAP) (Cenci et al., 2003); *moi* produces a protein that does not contain known functional motifs (Raffa et al., 2009); and *verrocchio (ver)* specifies a protein that contains an OB-fold domain structurally homologous to the STN1 OB-fold (Raffa et al., 2010). The HOAP, Ver, and Moi proteins directly interact with each other; HOAP and Moi also bind HP1 but Ver does not. An additional protein required to prevent telomere fusion, called HP1-HOAP-interacting protein (HipHop), was identified among the polypeptides that co-precipitate with HOAP (Gao et al., 2010). HOAP and HipHop appear to be mutually dependent for their stability. In HOAP-depleted cells HipHop is almost undetectable by Western blotting, and HipHop depletion causes a reduction of the HOAP level (Gao et al., 2010). It is currently unknown whether HipHop interacts with Moi and Ver.

**Table 1:** ***Drosophila* genes required to prevent telomere fusion in mitotic cells**.

Gene name	Protein name	TFs/cell in mutants	Function outside telomeres	Human homolog	Function at human telomere
*cav*	HOAP	5	None known	None	–
*hiphop*	HipHop	Many (1)	None known	None	–
*moi*	Moi	5	None known	None	–
*ver*	Ver	5	None known	STN1 (3)	–
*Su(var)205*	HP1α	4	Heterochromatin regulation; transcription factor	CBX5/ HP1α	Yes
*eff*	UbcD1	0.7	E2 ubiquitin-conjugating enzyme	UBE2D2/; UbcH5b	Nd
*woc*	Woc	2	Transcription factor	ZMYM3/; ZNF261	Nd
*mre11*	Mre11	0.5	DNA repair	MRE11	Yes
*rad50*	Rad50	0.5	DNA repair	RAD50	Yes
*nbs*	Nbs	0.4	DNA repair	NBS1	Yes
*tefu*	ATM	0.6	Kinase; DNA damage response	ATM	Yes
*mei-41*	ATR	None (2)	Kinase; DNA damage response	ATR	Yes
*mus-304*	ATRIP	None (2)	DNA helicase; DNA damage response	ATRIP	Yes

*(1) The frequency of TFs elicited by loss of HipHop has been determined by RNAi in S2 tissue culture cells and not in mutant brains. (2) Mutations in *mei-41* or *mus-304* do not cause TFs but enhance the TF frequency in a *tefu* mutant background so that *mei-41*, *tefu*, and *mus-304 tefu* double mutants exhibit TF frequencies that are much higher than those seen in *tefu* single mutants. (3) The Ver protein has only structural homology with the OB-fold domain of STN1. Nd, not determined*.

**Figure 2:**
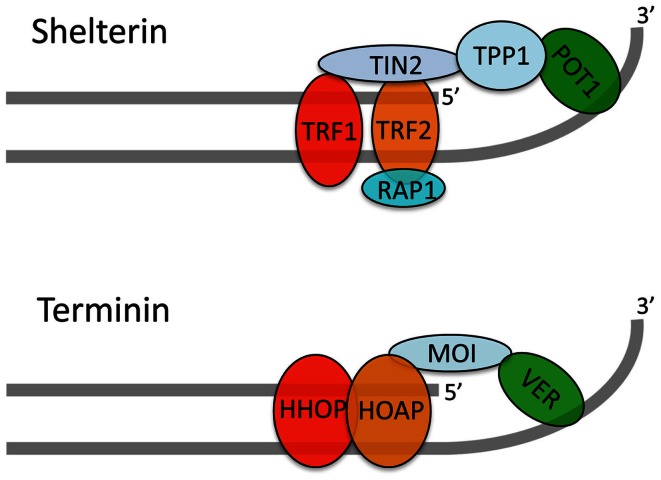
**A tentative model for the terminin structure**. Our published and unpublished results suggest that terminin and shelterin have similar architectures. We propose that HOAP and HipHop are primarily bound to the telomeric DNA duplex while Ver interacts with the single stranded overhang; Moi would connect HOAP/HipHop to Ver without binding DNA. It should be noted that direct evidence that *Drosophila* telomeres terminate with a single stranded overhang is still lacking. In addition, this overhang might not be present in all telomeres as suggested by early studies (Biessmann et al., 1990).

Immunolocalization experiments on both mitotic and polytene chromosomes have shown that HOAP and HipHop are exclusively enriched at telomeres, where they precisely co-localize (Cenci et al., 2003; Gao et al., 2010). An analysis of GFP-Moi and Ver-GFP localization on polytene chromosomes showed that these proteins are also enriched only at telomeres. However, GFP-Moi and Ver-GFP could not be detected at mitotic chromosome ends, probably due to their very low abundance (Raffa et al., 2009, 2011). HOAP and HipHop localize to the extremities of various types of TDCs demonstrating that these proteins bind chromosome ends in a sequence-independent fashion (Cenci et al., 2003; Gao et al., 2010; Titen and Golic, 2010). These results strongly suggest that HOAP, Moi, Ver, and HipHop form a complex, we call terminin, which specifically binds and protects *Drosophila* telomeres. The structural and functional characterization of the terminin complex is still incomplete and both the architecture and the individual roles of terminin subunits are poorly defined. However, the extant data indicate that HOAP and HipHop are primarily bound to the telomeric DNA duplex while Ver and Moi are associated with the single stranded overhang (Figure 2; Raffa et al., 2011). Collectively, the studies on terminin indicate that this complex specifically accumulates at telomeres throughout the cell cycle and functions only at telomeres. Thus, terminin has the same properties of shelterin, suggesting that the two complexes are functionally analogous (Raffa et al., 2009, 2010, 2011).

Similar to the shelterin subunits, individual terminin components do not play identical roles at *Drosophila* telomeres. Mutations in *cav* elicit both the DDR and the spindle assembly checkpoint (SAC), while mutations in *moi* or *ver* have little or no ability to trigger these checkpoints. However, *moi* and *ver* are essential to hide chromosome ends from the DNA repair machineries that mediate telomere fusion (Ciapponi and Cenci, 2008; Musarò et al., 2008; Cenci, 2009; Raffa et al., 2009, 2011). It should be noted that loss of HOAP destabilizes HipHop and prevents telomeric localization of Moi and Ver (Raffa et al., 2009, 2011; Gao et al., 2010). Thus, HOAP-depleted *Drosophila* telomeres lack all terminin components and are thus analogous to human telomeres lacking all shelterin subunits. Consistent with this analogy, shelterin- and terminin-free telomeres activate both the ATM and ATR/ATRIP signaling and are processed by NHEJ pathways leading to telomere fusion. The SAC response in terminin-free flies appears to be mediated by the BubR1 kinase, which accumulates at the uncapped telomeres in *cav* mutant cells. It has been shown that dysfunctional telomeres of TRF1-overexpressing mice also recruit BubR1, but it is unclear whether telomere-associated BubR1 can activate the SAC response (Muñoz et al., 2009).

## Non-Shelterin Proteins Involved in Human Telomere Maintenance

The shelterin subunits interact with several conserved proteins, often called shelterin accessory factors (Palm and de Lange, 2008), which are also required for telomere maintenance. These proteins include several DNA repair factors such as the ATM and Chk2 kinases, the Ku70/80 heterodimer, the MRE11-RAD50-NBS1 (MRN) complex, Rad51, the ERCC1-XPF, and MUS81 endonucleases, the Apollo exonuclease, the RecQ family members WRN and BLM, and the RTEL1 helicase. In addition, human telomeres are enriched in the ORC and CST complexes, HP1 homologs, and the SUV39 histone methyltransferase (reviewed by Palm and de Lange, 2008; Martinez et al., 2009; Jain and Cooper, 2010). Loss of these shelterin accessory factors results in diverse telomere phenotypes. For example, loss of Ku70/80 results in frequent TFs, while inhibition of the Apollo, BLM, RTEL1, or CST function disrupt telomere replication (Palm and de Lange, 2008; Sfeir et al., 2009; Ye et al., 2010; Gu et al., 2012; Vannier et al., 2012). Despite the similarity in the phenotypes they elicit, shelterin accessory factors and shelterin subunits do not share the same properties, as the accessory factors do not localize and do not function exclusively at telomeres.

## Non-Terminin Proteins Required for *Drosophila* Telomere Protection

In addition to the terminin components, studies carried out in the past 15 years identified nine proteins directly or indirectly required to prevent telomere fusion: HP1, Mre11, Rad50, Nbs, ATM, Mei-41/ATR, Mus-304/ATRIP, Eff/UbcD1, and Woc (Table 1). Unlike the terminin subunits these proteins do not localize and function only at telomeres but have multiple roles in diverse cell compartments. HP1 provides a paradigm for the non-terminin proteins. HP1 directly interacts with HOAP, HipHop, and Moi and precisely co-localize with terminin at chromosome ends. However, HP1 is not only enriched at the telomeres of polytene chromosomes, but also at the chromocenter, the fourth chromosome and many euchromatic bands (reviewed in Fanti and Pimpinelli, 2008). Consistent with this localization pattern, in addition to telomere capping, HP1 is involved in a variety of processes including the maintenance of proper chromatin structure, DNA replication and repair, transcriptional regulation, and gene silencing (Fanti et al., 1998; Fanti and Pimpinelli, 2008; Piacentini et al., 2009; Vermaak and Malik, 2009; Chiolo et al., 2010).

The non-terminin proteins required to prevent telomere fusion include four factors involved in DNA repair: Mre11, Rad50, and Nbs that form the conserved MRN complex, and the ATM kinase encoded by *telomere fusion (tefu)* gene (Bi et al., 2004, 2005; Ciapponi et al., 2004, 2006; Oikemus et al., 2004, 2006; Silva et al., 2004; Song et al., 2004). Mutations in the ATR-encoding *mei-41* gene or in the *mus-304* gene that encodes the ATR-interacting protein ATRIP do not cause TFs, but interact with mutations in *tefu*, so that the *tefu mei-41* and *tefu mus-304* double mutants exhibit dramatic increases in TFs compared with *tefu* single mutants (Bi et al., 2005). It is currently unknown whether the MRN subunits or ATM physically interact with terminin. However, mutations in the *rad50*, *mre11*, and *nbs* genes strongly reduce HOAP and Moi accumulation at telomeres. Mutations in *tefu*, *mei-41*, or *mus-304* have little or no effect on HOAP localization at mitotic telomeres but *tefu mei-41* and *tefu mus-304* fail to recruit HOAP at chromosome ends (reviewed in Rong, 2008; Raffa et al., 2011). These results indicate that terminin recruitment at telomeres requires the wild type function of the MRN complex and the function of either ATM or ATR. Thus, ATM and ATR/ATRIP have partially redundant roles in telomere protection suggesting that failure to phosphorylate a common but as yet unknown target leads to deprotected telomeres. The mechanism by which the combined action of the MRN complex, ATM, and ATR-ATRIP leads to terminin recruitment to telomeres is unclear. It has been suggested that interactions of the DNA ends with these DNA repair proteins result in conformational changes that facilitate terminin recruitment (reviewed in Ciapponi and Cenci, 2008; Rong, 2008).

Effete/UbcD1 is a highly conserved E2 ubiquitin-conjugating enzyme implicated in several *Drosophila* cellular processes (Cenci et al., 1997). The Eff protein is a major constituent of Drosophila chromatin with repressive properties (Filion et al., 2011), and is enriched at many polytene chromosome bands (Cipressa and Cenci, unpublished observations). However, the telomere-associated target(s) of Eff remain to be identified. Given that inactivation of the proteasome does not cause TFs (our unpublished results), Eff-mediated ubiquitination is probably not aimed at protein degradation but is instead a post-translational modification that ensures proper capping function of one or more telomere-associated proteins. Polytene chromosomes from *eff* mutants exhibit normal amount of HOAP, suggesting that *eff* function is not required for terminin recruitment and or maintenance at telomeres (reviewed in Raffa et al., 2011).

*without children (woc*) gene encodes a zinc finger protein that interacts with HP1c and functions both in transcriptional regulation and telomere capping (Raffa et al., 2005; Font-Burgada et al., 2008). Woc co-localizes with the initiating form of Pol II in many euchromatic bands and is also enriched at telomeres. Woc localization at telomeres is not affected by *cav* mutations and mutations in *woc* do not affect HOAP localization at chromosome ends (Raffa et al., 2005). These results indicate that the Woc function at telomeres is independent of that played by HOAP. It remains to be determined whether the Woc function is also independent of those played by Moi and Ver.

In summary, the nine non-terminin proteins required for telomere capping in *Drosophila* brains are all conserved in humans. In addition, there is evidence the human homologs of HP1, Mre11, Rad50, Nbs, Tefu/ATM, Mei-41/ATR, and Mus-304/ATRIP associate with human telomeres and play telomere-related functions. Eff/UbcD1 and Woc are also conserved but it is currently unknown whether their human counterparts have roles at telomeres.

## *Drosophila* Telomeres as Model to Detect New Proteins Involved in Human Telomere Maintenance

While the non-terminin proteins are evolutionarily conserved, none of the terminin proteins, with the possible exception of Ver, has homologs in yeasts, mammals or plants. In addition, all terminin proteins exhibit very high rates of non-synonymous substitution per non-synonymous site, and are therefore fast-evolving proteins; non-terminin proteins are not fast-evolving and exhibit relatively low non-synonymous substitution rates (Gao et al., 2010; Raffa et al., 2010, 2011). Based on these results, we hypothesized that following telomerase loss, *Drosophila* lost the shelterin and the CST homologs that bind DNA in a sequence-specific fashion, and evolved terminin to bind chromosome ends independently of the DNA sequence. It is indeed conceivable that the transition from a telomerase-driven to a transposon-driven telomere elongation mechanism resulted in a divergence of terminal DNA sequences, accompanied by a strong selective pressure toward the evolution of sequence-independent telomere-binding factors. We also hypothesized that the non-terminin proteins did not evolve as rapidly as terminin because of the functional constraints imposed by their involvement in diverse cellular processes (Raffa et al., 2009, 2010, 2011).

Our hypothesis on terminin evolution suggests that non-terminin proteins correspond to ancestral telomere-associated proteins. Indeed, of the nine non-terminin proteins so far identified, seven are implicated in human telomere maintenance. Thus, it appears that the main difference between *Drosophila* and human telomeres is in the protective complexes that specifically associate with the DNA termini. It has been estimated that the *Drosophila* genome contains at least 40 genes required to prevent telomere fusion (Cenci et al., 2005). We believe that the identification of additional *Drosophila* genes encoding non-terminin proteins involved in telomere protection will lead to the discovery of novel human telomere components.

## Conflict of Interest Statement

The authors declare that the research was conducted in the absence of any commercial or financial relationships that could be construed as a potential conflict of interest.
